# Schema-related cognitive load influences performance, speech, and physiology in a dual-task setting: A continuous multi-measure approach

**DOI:** 10.1186/s41235-018-0138-z

**Published:** 2018-12-07

**Authors:** Maria Wirzberger, Robert Herms, Shirin Esmaeili Bijarsari, Maximilian Eibl, Günter Daniel Rey

**Affiliations:** 1 Psychology of Learning with Digital Media, Institute for Media Research, Faculty of Humanities, TU Chemnitz, Straße der Nationen 12, 09111 Chemnitz, Germany; 2 Media Informatics, Faculty of Computer Science, TU Chemnitz, Straße der Nationen 62, 09111 Chemnitz, Germany

**Keywords:** Schema acquisition, Cognitive load assessment, Dual-task setting, Prosodic speech parameters, Physiological measures

## Abstract

Schema acquisition processes comprise an essential source of cognitive demands in learning situations. To shed light on related mechanisms and influencing factors, this study applied a continuous multi-measure approach for cognitive load assessment. In a dual-task setting, a sample of 123 student participants learned visually presented symbol combinations with one of two levels of complexity while memorizing auditorily presented number sequences. Learners’ cognitive load during the learning task was addressed by secondary task performance, prosodic speech parameters (pauses, articulation rate), and physiological markers (heart rate, skin conductance response). While results revealed increasing primary and secondary task performance over the trials, decreases in speech and physiological parameters indicated a reduction in the overall level of cognitive load with task progression. In addition, the robustness of the acquired schemata was confirmed by a transfer task that required participants to apply the obtained symbol combinations. Taken together, the observed pattern of evidence supports the idea of a logarithmically decreasing progression of cognitive load with increasing schema acquisition, and further hints on robust and stable transfer performance, even under enhanced transfer demands. Finally, theoretical and practical consequences consider evidence on desirable difficulties in learning as well as the potential of multimodal cognitive load detection in learning applications.

## Significance

Interactive learning and training technologies enhance task opportunities in various application domains but may also increase demands on cognitive resources. Arising pitfalls can be avoided by providing systems with knowledge about users’ current mental states, which allows regulating system responses in an adaptive and personalized way. For instance, during learning activities, a system could align features such as the amount and speed of the presented content or the degree of instructional support to increase motivation, encourage sustained performance, and foster system acceptance. Related challenges firstly involve measurement issues, i.e., an accurate user state recognition that requires intelligent algorithms for correctly interpreting patterns contained in the acquired signals. Secondly, the system behavior needs to be continuously adjusted to meet users’ needs in the most sophisticated way possible. The current research provides relevant pre-requisites for both issues by monitoring variations in cognitive demands over the task with a novel combination of sensitive measures related to performance, speech, and physiological reactions. Applying the summarized evidence to real-world applications, dynamic recognition algorithms could be developed for computerized learning devices such as mobile systems that incorporate wearable multimodal sensors.

## Background

Looking back in the history of cognitive psychology, there is a long research tradition on cognitive schemata as crucial outcomes of learning processes (Ghosh & Gilboa, 2014). Once knowledge has been acquired successfully, it is represented and organized in small bundles of information that are constructed during learning and applied automatically in later process stages. Research in this field has mainly covered structural aspects (Bartlett, 1932; Rumelhart, 1980) and mechanisms of schema acquisition and adjustment (Piaget, 1952), but less concern was devoted to related demands on learners’ cognitive resources and their changes during the learning process. The current study addresses this research gap by monitoring the interplay of load inducing factors in a controlled learning scenario with a combination of continuous cognitive load measures.

### Theoretical perspectives in cognitive load research

As we consider cognitive resource investment in instructional situations, the Cognitive Load Theory (CLT; Sweller, Van Merriënboer, & Paas, 1998; Sweller, Ayres, & Kalyuga, 2011) becomes an indispensable source of explanation. In brief, the theory resides on three core assumptions: Firstly, based on well-established memory models (Anderson, 1983; Atkinson & Shiffrin, 1971; Baddeley, 1992), it postulates limited working memory resources in terms of duration and capacity of information storage. Secondly, long-term memory resources are assumed to lack such boundaries and hold benefits for elaborated learning processes. Thirdly, mental representation of knowledge should occur via schemata, described as organized knowledge structures with stable patterns of relationships between elements (Kalyuga, 2010). A further characteristic of the theoretical framework is the separation of the overall cognitive load construct into the facets of intrinsic, extraneous, and germane cognitive load (Sweller et al., 1998). Whereas intrinsic cognitive load (ICL) results from the number of interrelated elements of information, determining the complexity of the used learning material relative to learners’ previous knowledge, extraneous cognitive load (ECL) is associated with the surrounding instructional situation, i.e., ways of content presentation or situational constraints. Germane cognitive load (GCL) arises from relevant processes of schema acquisition and automation. Prior research shows that high levels of ECL hamper learning performance, but only if high amounts of ICL are present at the same time (Sweller et al., 1998). Whereas ECL should be minimized and ICL kept at a manageable level, the instructional focus is put on fostering GCL to achieve optimal learning outcomes.

Theory-related discussions in the more recent past addressed the assumed additive relationship between ICL, ECL, and GCL (De Jong, 2010; Park, 2010; Sonnenfeld & Keebler, 2016) as well as substantial redundancies in the facet of GCL. This facet was introduced in addition to the initial two-component framework mainly on theoretical accounts instead of empirical evidence (Sweller et al., 1998). These issues resulted in efforts on reformulating the postulated theoretical framework. One approach suggests a reduction of cognitive load facets back into a two-component model, which contrasts productive factors beneficial for learning (ICL) with unproductive factors that impair learning (ECL) and subsumes GCL under the facet of ICL (Kalyuga, 2011; Kalyuga & Singh, 2016; Sweller, 2010). Another approach postulates a process-driven reconceptualization of the three-component model that quantifies temporal changes in GCL over the task (De Jong, 2010; Sonnenfeld & Keebler, 2016; Wirzberger, Esmaeili Bijarsari, & Rey, 2017). Following this view, ICL, ECL, and GCL reside at different levels of inspection: a structural level in terms of ICL and ECL, which can be determined a priori (Beckmann, 2010; Wirzberger, Beege, Schneider, Nebel, & Rey, 2016), and a processual level in terms of GCL, which undergoes changes throughout the learning task and depends on the achieved level of schema acquisition.

Empirical evidence for the latter approach arises from Wirzberger et al. (2017), who applied a basal learning task that a priori varied the amount of interacting information elements (ICL) and induced interruptions at several points over the task (ECL). Statistical analyses compared linear, quadratic, and logarithmic progression models, and the results suggested a logarithmic progression of schema-related cognitive load (GCL) over time, influenced by structural features. The resulting inversion of the learning curve (Ebbinghaus, 1964) aligns well with established evidence on cognitive skill acquisition (Anderson, 1983; Kraiger, Ford, & Salas, 1993; Shiffrin & Schneider, 1977). It also provides further evidence that building and organizing schematic structures of knowledge in the initial process stages shed higher demands on cognitive resources, whereas automation and tuning procedures in later process stages require smaller resource supplies. Although the approach already yielded promising results, the study raised the need for a more continuous controlled inspection of the determined mechanisms.

Related questions on the durability and robustness of previously established schemata might be addressable by applying acquired knowledge structures on distinct but related problems. Kalyuga (2010) particularly recommends tasks involving grouping or categorizing to create such transfer demands. Evidence on expertise development shows that novice learners require less complex tasks in early training stages to engage in robust and stable schema acquisition (Van Merriënboer, Kester, & Paas, 2006); thus, novice learners dealing with a complex task right from the beginning should perform worse. In addition, extended transfer requirements would overly demand cognitive resources and further decline performance.

### Approaches to cognitive load assessment

Studying the assumed interplay of cognitive load factors requires their valid assessment. Several efforts have been made, relating to performance, psychophysiology, behavior, and self-report (Sweller et al., 2011; Wickens, Hollands, Banbury, & Parasuraman, 2013; Zheng, 2018). From the variety of approaches, a selection will be discussed in the following, which is considered to be relevant for the applied task framework.

Since learners’ performance is explicitly addressed and recorded in learning scenarios, the inspection of performance-related parameters offers valuable insights. Such measures usually operate on observable performance indices in dual-task paradigms that use secondary tasks to induce and/or assess cognitive load (O’Donnell & Eggemeier, 1986). If the secondary task mainly serves to induce cognitive load, primary task performance is observed, whereas the inspection of secondary task performance is applied for purposes of assessing cognitive load (Brünken, Plass, & Leutner, 2004; Brünken, Steinbacher, Plass, & Leutner, 2002; Kraiger et al., 1993; Park & Brünken, 2018). A conjoint observation of both aspects provides a more comprehensive view; thus, often both parameters are inspected complementarily. In terms of task-related stimulus and response modalities, evidence on modality compatibility in dual-task performance shows that for spatial codes a combination of visual input and manual output is superior to auditory input and vocal output, whereas the results are reversed for verbal codes (Hazeltine, Ruthruff, & Remington, 2006). For this reason, when employing dual-task techniques, compatible input and output modalities for both tasks should be used so that resource interference only occurs due to content-related processing demands. Participants’ performance in primary and secondary tasks can further be evaluated in terms of efficiency (Hoffman & Schraw, 2010), with higher levels of efficiency corresponding to high performance and low effort. Efficiency measures usually are calculated from effort indicators like response time, which indicates how cognitively demanding a task was, and performance indicators like correct responses (Sweller et al., 2011).

Bodily functions are often affected involuntarily when people are put under cognitive demands and thus provide reliable online indicators of current levels of cognitive load. Among the variety of psychophysiological techniques, heart rate (HR) and skin conductance response (SCR) have already shown sensitivity to changes in cognitive resource demands in dual-task settings (Mehler, Reimer, & Coughlin, 2012). Both parameters indicate increasing levels of imposed cognitive load by increasing values.

Besides involuntarily occurring bodily responses, participants show voluntarily behavioral reactions as well. The effects of cognitive load on duration-based speech parameters can be classified into the field of prosody, for instance, disfluency, articulation rate, content quality, the number of syllables, and silent pauses as well as filled pauses (Berthold & Jameson, 1999; Müller, Großmann-Hutter, Jameson, Rummer, & Wittig, 2001). Evidence suggests that, with increasing levels of cognitive load, speaking rates (the number of syllables per time) and articulation rates (the number of syllables per time excluding pauses) decrease. More and longer pauses during speech flow, induced by planning processes, also reflect higher levels of cognitive load (Esposito, Stejskal, Smékal, & Bourbakis, 2007; Khawaja, Ruiz, & Chen, 2007, 2008; Müller et al., 2001). So far, the described speech parameters have been applied to determine cognitive load in task settings that demand cognitive resources on a shorter time span, for instance, a reading span task or a Stroop interference task under time pressure (Yap, 2012). A novel perspective arises by applying this approach to capture naturally occurring changes in cognitive resource demands due to schema acquisition processes.

Beneath the introduced objective measures, subjective means of assessment can be applied as well. Self-report rating scales comprise an easily applicable and widely used approach in cognitive load assessment that relies on learners’ ability to provide valid retrospective estimations of the experienced level of cognitive load. A rating scale that addresses the facets of ICL, ECL, and GCL independently was developed by Leppink, Paas, Van der Vleuten, Van Gog, and Van Merriënboer (2013). Higher levels of subjectively experienced cognitive resource demands on each facet are indicated by higher ratings on the related items.

In summary, each measure entails certain strengths and weaknesses (Martin, 2018; Sweller et al., 2011; Wickens et al., 2013). Although performance-related parameters provide a continuous assessment and often emerge directly from the task, secondary tasks potentially interfere with primary tasks and require thoughts regarding the adequate level of complexity as well as the employed stimulus and response modalities. Psychophysiological techniques provide a continuous and reliable assessment, since physiological responses are hardly controllable voluntarily, but these techniques require special equipment, substantial expertise, and effort in application and analysis to avoid and control for artifacts and noise. Behavioral parameters also ensure a continuous and reliable way of assessment but require high expertise and effort as well. While subjective ratings via questionnaires are rather easily applicable, they provide no continuous assessment and rely on participants’ retrospective estimations of prior resource demands. For handling the outlined limitations, a combination of different assessment approaches is therefore regarded as the most promising solution to strengthen the informative value of the emerging results (Chen, Zhou, & Yu, 2018; Korbach, Brünken, & Park, 2018).

### Present experiment

To address the outlined research gaps, the current study focused on changes in cognitive load related to the process of schema acquisition, extended by the issue of robustness of the obtained schemata. A basal learning task and a related basal schema application task (Kalyuga, 2010) provided a concise, controllable, and internally valid framework. By combining a set of continuous performance-related, behavioral, and physiological measures, completed by subjective self-reports, the comprehensive capture of underlying cognitive processes was ensured.

### Hypotheses

Based on the introduced theoretical background, a logarithmically decreasing level of cognitive load with increasing schema acquisition was expected, which should be observable in performance, speech, and physiological parameters (*hypothesis 1a*). Moreover, an increase in subjectively reported cognitive load with a higher level of task complexity was postulated (*hypothesis 1b*). Regarding retention and transfer performance, with a higher level of task complexity a decrease in retention performance (*hypothesis 2a*) and transfer performance (*hypothesis 2b*) was assumed, as well as a decrease in transfer performance with increasing transfer demands (*hypothesis 2c*).

## Methods

### Pre-study

A schema application task was designed to address the robustness of participants’ schema acquisition. It required participants to categorize symbol combinations with reference to a displayed target symbol as either correct or false, according to previously acquired knowledge. Combinations were composed from the four geometrical symbols square, star, triangle, and circle. Some symbol combinations included non-prototypical symbols to determine if the established schemata were stable enough to deal with such kind of transformation. A pre-study should ensure that non-prototypical symbols are still categorized according to their underlying prototype.

#### Pre-study methods

Seventy-four participants (*M*_age_ = 35.00 years, *SD*_age_ = 13.09, range 19–64, 63.51% female) completed the test. Of these, 54.05% had already graduated or were currently enrolled as graduate students, 20.27% had completed an apprenticeship, 10.81% were undergraduate students, and the remaining 14.87% reported diverse levels of graduation or did not reveal their educational status.

**Fig. 1:**
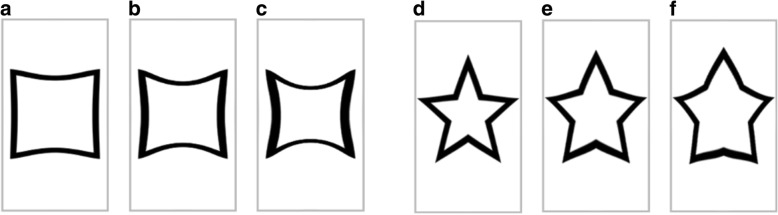
Distorted symbols. **a** = square 30, **b** = square 60, **c** = square 90, **d** = star − 30, **e** = star − 60, **f** = star − 90

Non-prototypical symbols were generated with Adobe Photoshop and comprised different severities of either barrel (outward, negative deviation from 0) or pincushion (inward, positive deviation from 0) distortion. As displayed in Fig. 1, the levels of distortion ranged from small (± 30%) to medium (± 60%) to high (± 90%).

Participants filled out an online questionnaire including the prototypical and non-prototypical symbols accompanied with a categorization request. After classifying each symbol as either circle, square, star, or triangle, participants had to rate the level of distortion on a seven-point Likert scale with verbal anchoring at the extreme points to determine the perceived severity of deviation from the underlying prototype. As the prototypes were included in the presented symbol set as well, the scale started from 0 (“not at all”) and reached 6 (“very strong”) to provide participants the opportunity of a valid and reliable rating on a sufficient level of complexity (Lozano, García-Cueto, & Muñiz, 2008).

#### Pre-study results

Descriptive analyses of categorization frequencies and distortion ratings are reported in Table 1. They indicate rather stable and homogeneous classifications of non-prototypical symbols according to their underlying prototype, with at least 94.7% correct categorizations, even on the broader distribution of age and educational backgrounds. Moreover, even on a descriptive level, distortion ratings outline an increasing amount of perceived deviation with increasing severity of distortion.

**Table 1:** Categorization and distortion rating of prototypical and distorted symbols

Dist.	Circle			Square			Star			Triangle		
	%	*M*	*SD*	%	*M*	*SD*	%	*M*	*SD*	%	*M*	*SD*
0	100	1.09	0.38	100	1.12	0.72	97.3	1.03	0.16	100	1.03	0.16
– 30	100	2.45	1.04	100	2.82	1.03	100	1.49	0.82	100	2.28	0.77
30	98.7	2.20	0.98	98.7	2.76	1.07	100	1.27	0.45	100	2.81	1.18
– 60	100	3.27	1.31	98.7	3.77	1.17	100	2.14	1.24	98.7	3.14	1.16
60	100	3.18	1.33	100	3.72	1.28	100	2.08	1.16	94.7	4.03	1.24
– 90	100	3.69	1.33	100	4.65	1.21	100	2.32	1.25	100	3.46	1.28
90	98.7	3.70	1.32	97.3	4.53	1.40	98.7	3.61	1.50	98.7	4.59	1.32

*Dist.* severity of outward or inward distortion, *%* percentage of correct categorizations, *M* mean of distortion rating, *SD* standard deviation of distortion rating

Repeated measures analyses of variance (ANOVAs) on the distortion ratings were computed separately for each symbol category, with severity of distortion as a sevenfold within-subjects factor. Mauchly’s test indicated a violation of sphericity for all symbol categories; therefore, the degrees of freedom were corrected using Greenhouse-Geisser estimates for circle, χ^2^(20) = 69.184, *p* < .001, ε = 0.751, square, χ^2^(20) = 61.139, *p* < .001, ε = 0.778, star, χ^2^(20) = 156.934, *p* < .001, ε = 0.608, and triangle, χ^2^(20) = 49.509, *p* < .001, ε = 0.837. The results indicated strong and highly significant main effects of distortion severity for circle, *F*(4.504, 388.761) = 109.263, *p* < .001, η_p_^2^ = .599, square, *F*(4.668, 340.729) = 151.472, *p* < .001, η_p_^2^ = .675, star, *F*(3.649, 266.373) = 87.503, *p* < .001, η_p_^2^ = .545, and triangle, *F*(5.020, 366.476) = 175.157, *p* < .001, η_p_^2^ = .706.

Post hoc pairwise comparisons with the Bonferroni-Holm correction (Maxwell, 1980) revealed significant differences between distorted symbols and prototypes for all levels of distortion severity in each symbol category (*p* < .001). When comparing outward and inward distortion within and across distortion severities, diverse patterns appeared depending on the symbol category. For both circle and square, no significant differences resulted between 30 vs. − 30, 60 vs. − 60, and 90 vs. − 90, whereas all other comparisons achieved significance with at least *p* < .05. In the star category, no significant differences resulted between 60 vs. – 60, 60 vs. 90, and − 60 vs. 90, but significant differences (*p* < .001) occurred for all other comparisons. All pairwise comparisons achieved significance in the triangle category with at least *p* < .05.

Based on both categorization frequencies and distortion ratings, non-prototypical symbols with superior psychometric properties were chosen. In addition to considering correct classifications and significant pairwise comparisons, a balanced representation of both barrel and pincushion distortion across symbols was sought. The resulting pattern comprised − 30, − 60, − 90 for circle, − 30, 60, − 90 for square, 30, 60, − 90 for star, and 30, − 60, − 90 for triangle.

### Main study

#### Participants

A total of 123 undergraduate and graduate students (*M*_age_ = 22.67 years, *SD*_age_ = 3.55, range 18–34, 76.42% female) participated in the main study. They were enrolled in Communication Science (41.32%), Psychology (30.58%), Science, Technology, Engineering, and Mathematics (STEM) fields (11.57%), Humanities (9.09%), or Education (5.79%) and received either a financial allowance of 5€ (49.59%) or course credits (50.41%) as compensation. Experimental conditions did not differ regarding age, *t*(119.05) = 0.62, *p* = .539, *d* = 0.111, gender, χ^2^(1) < 0.01, *p* = .960, distribution of study courses, χ^2^(6) = 4.42, *p* = .620, or compensation choice, χ^2^(1) = 0.40, *p* = .527.

#### Design

The chosen learning task required participants to detect, remember, and retrieve four easy or difficult combinations of arbitrary geometric symbols while simultaneously memorizing five-digit number sequences as a secondary task. The resulting experimental design included task complexity as an independent between-subjects factor that varied due to the arrangement of the symbol combinations (three vs. four symbols, symbol order). As dependent variables, learners’ performance in both primary and secondary task, spoken responses on the secondary task, and physiological reactions were recorded continuously during the learning task. The standardized cognitive load questionnaire by Leppink et al. (2013) provided a summative evaluation of the inspected cognitive load facets. An open question on schema recall after the learning task enabled insights into the quality of schema acquisition over the task. Participants’ working memory capacity was derived from a translated version of the automated operation span task (OSPAN; Unsworth, Heitz, Schrock, & Engle, 2005) and used as the control variable.

With reference to the CLT, task complexity reflected the ICL component and was addressed according to the concept of element interactivity (Sweller, 2010). Unlike the work of Wirzberger et al. (2017), the scope of symbol combinations was increased by one element to avoid effects of boredom due to insufficiently low levels of task demands. The embedded secondary task characterized the ECL component, which aligns with the conceptualization of ECL as situational constraints (Wickens et al., 2013). Furthermore, the combined inspection of the continuous cognitive load measures hinted at the underlying cognitive resource investment pattern and represented the GCL component (Sweller et al., 2011). Whereas primary task efficiency resources were invested in schema acquisition relative to the achieved outcome (Hoffman & Schraw, 2010; Paas & Van Gog, 2006), secondary task efficiency hinted at the actual load imposed by the primary task. Based on this view, available resources would arise only due to already established and usable schemata (Kraiger et al., 1993).

An additional task on applying the obtained schemata by solving a categorization task addressed the robustness of the schema acquisition process. It required participants to evaluate the correct match of displayed input and response parts of the previously acquired symbol combinations, which included distorted symbols in parts of the trials. The underlying 2 × 5 factorial mixed design included a between-subjects factor task complexity (easy vs. difficult) and a within-subjects factor level of distortion (0 vs. 30 vs. 60 vs. 90) as independent variables. Reaction time and a corrected error score were recorded as dependent variables.

#### Materials

##### Learning task

Computer-based tasks were realized with OpenSesame (Mathôt, Schreij, & Theeuwes, 2012) and provided on a standard desktop computer with a 24″ monitor. Trials within the learning task were inspired by the procedure of the OSPAN (Unsworth et al., 2005) and comparable automated complex span tasks, which include a distractor task and a target task in each trial in alternating sequence. In the current study, within each trial the number task (secondary task) was presented first as a distractor and followed by the symbol task (primary task) as a target. Unlike the complex span task procedure, which involved new items to memorize in each trial for both the target and distractor tasks, the learning content of the primary task persisted during the entire task.

**Fig. 2:**
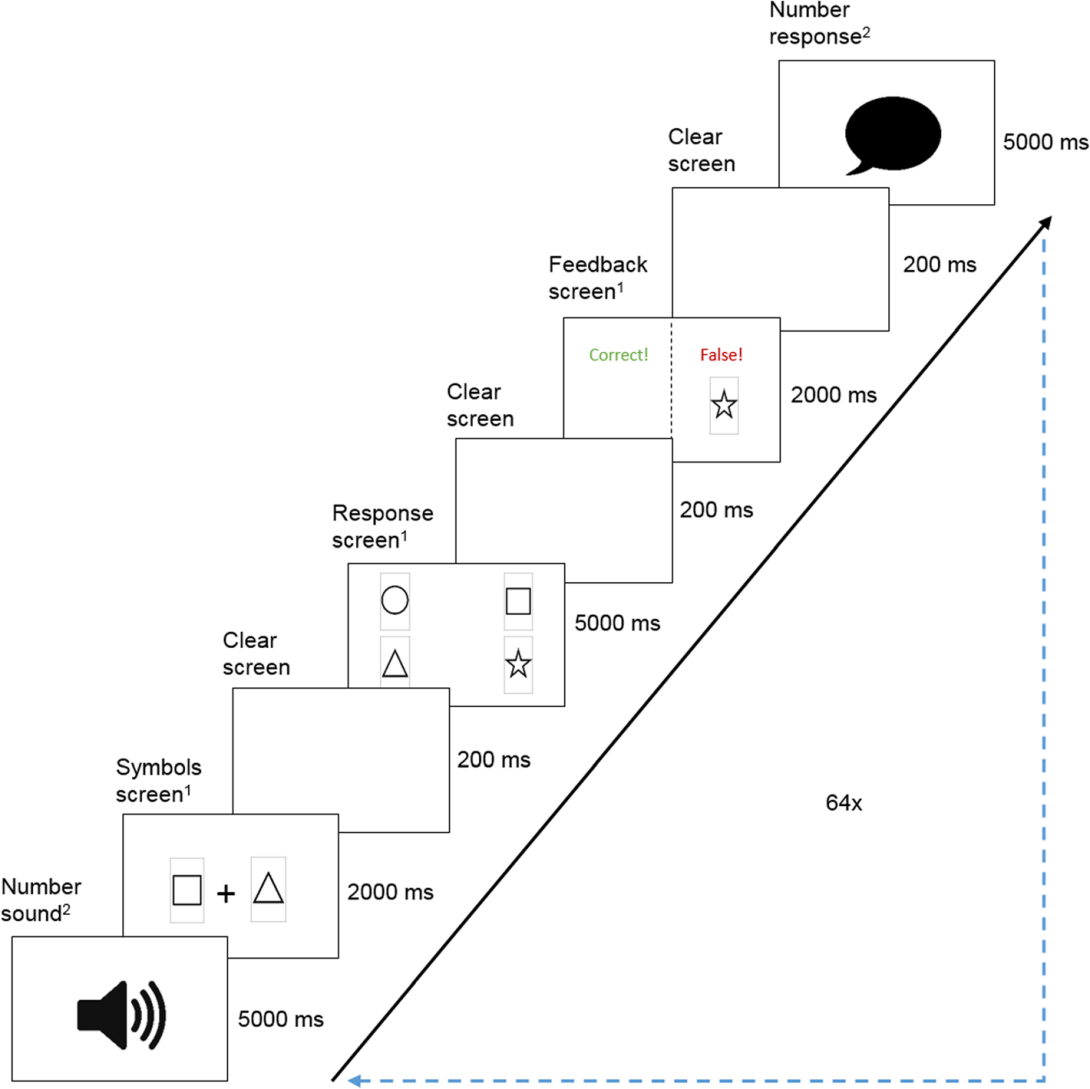
Schematic outline of the trial procedure in the learning task. Superscript indices indicate affiliation with the primary^1^ or secondary^2^ task

As outlined in Fig. 2, each of the 64 trials started with the auditory presentation of a unique randomly chosen five-digit number for 5000 ms. The amount of five digits, as well as the used time spans, was determined within a short internal pre-test with *N* = 7 participants (*M*_age_ = 28.71, *SD*_age_ = 2.43, range = 26–32, 4 male) and chosen to avoid distraction effects on the primary task by an overly complex secondary task. Indicated by a black speaker symbol on the screen, participants had to listen carefully and memorize the numbers in correct sequence. Afterward, a randomly chosen input part of one out of four abstract symbol combinations was presented for 2 s. This input part comprised two symbols (easy condition)  or three symbols (difficult condition). Participants had to remember the shown symbols in correct order and complete the sequence by choosing a symbol as a response on the next screen by mouse click. In this vein, a total of three (easy condition) or four (difficult condition) symbols formed a complete combination. As shown in Fig. 2, the response screen simultaneously presented the four possible response symbols in a randomly arranged 2 × 2 grid for 5 s. This was followed by a feedback screen lasting 2 s that also included the correct choice for false responses to foster correct schema acquisition. Finally, indicated by a black speech bubble symbol, participants had to recall the memorized digit sequence from the trial outset verbally within 5 s.

##### Schema application task

**Fig. 3:**
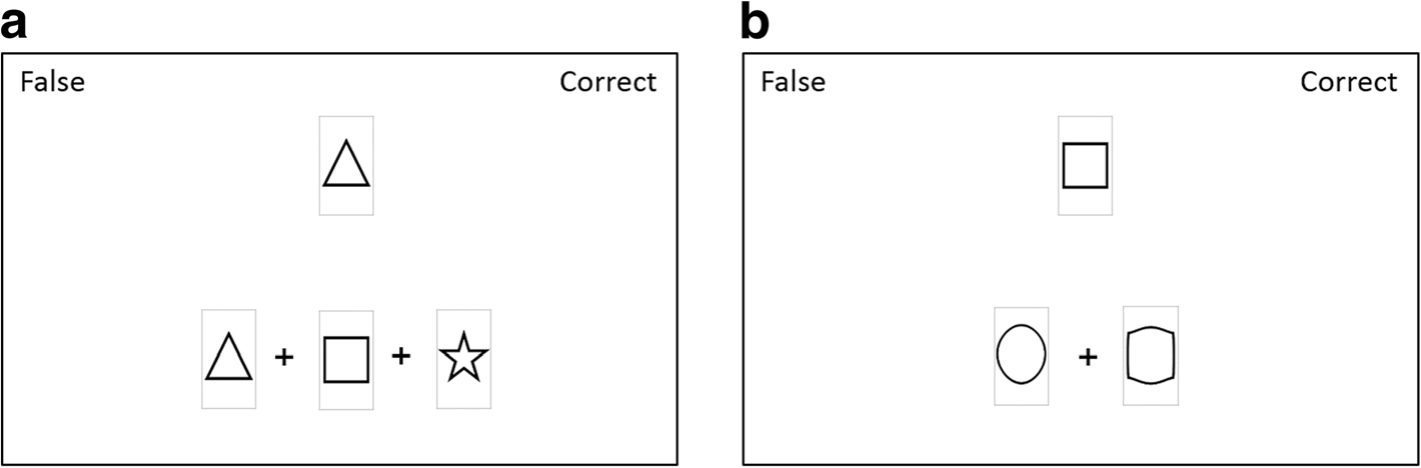
Stimuli in the schema application task: **a** difficult condition with prototypical symbols and **b** easy condition with distorted symbols

During each of the 60 trials in the schema application task, participants evaluated if the input part of a presented symbol combination matched or mismatched the response part. As depicted in Fig. 3, the response part of the symbol combination was shown in the upper part and the potential input part in the lower part of the screen. Response parts were always represented in prototypical symbols, whereas half of the input parts included distorted symbols from the pre-study. The pool of presented stimuli comprised correctly matched input and target parts, existing input parts with mismatched target parts, and non-existing input parts with mismatched target parts. Within 5 s, participants had to classify the presented combination as false or correct by pressing either the left (false) or right (correct) mouse key. Contrary to the learning task, participants received no further feedback on their response.

##### Questionnaires on retention and cognitive load

For assessing retention performance, participants received a computer-based form with a grid of three (easy condition) or four (difficult condition) empty boxes per combination in four rows. They had to recall the memorized symbol combinations by dragging symbols from an infinite pool for each possible symbol (i.e., circle, square, triangle, or star) via mouse click and dropping them into the existing grid boxes to form combinations. In addition, they could indicate corrections on the provided combinations in a separate comment space below. Participants’ subjectively perceived level of cognitive load was addressed on 11-point Likert scales via the 10-item questionnaire by Leppink et al. (2013) that differs between the three subscales of ICL, ECL, and GCL.

#### Procedure

Data were obtained in individual testing sessions of about 60 min in a separate laboratory, equipped with a standard desktop computer for the participant and an experimenter netbook to record the physiological data. At the outset of each testing session, participants were welcomed and signed an informed consent. This form outlined the purpose and procedure of the study and ensured that participants’ treatment aligned with approved ethical standards and that their privacy was respected. Afterward, the OSPAN had to be completed, which usually took about 15 min. Before starting the learning task, the experimenter had to fulfill several preparatory duties, requiring about 5 min. First, the electrodes for the physiological measures were attached to the left hand and a close-talk microphone for the speech recording was placed at the upper part of the participant’s sternum. Following an initial adjustment of the speech recording volume to individual voice characteristics, the learning task was completed within an average duration of 20 min. After the electrodes were removed, participants worked on the questionnaires on retention, cognitive load, and demographics, which were provided online and were completed in about 10 min by most participants. Finally, they completed the schema application task (about 10 min) and were debriefed and approved.

#### Scoring

*Primary task efficiency* was computed, following the likelihood model (Hoffman & Schraw, 2010), as the quotient of correct responses (performance) and reaction times (effort) within each trial. Since reaction times were retrieved in milliseconds, scores were multiplied by 1000 to obtain the proportion of correct responses per second. The resulting values provide a hint on the investment of available mental resources over the task: if learners start to perform faster and less erroneously on the task, they must invest less mental capacity.

For *secondary task efficiency*, also based on the likelihood model approach (Hoffman & Schraw, 2010), performance was computed by comparing spoken words to correct words from the reference and subtracting the amount of substituted, deleted, and inserted words (Lee, 1988) relative to the total number of words in the reference (word accuracy). The participant’s effort was reflected in the time starting from the presentation of the visual stimulus to the end of the last uttered digit (verbal response duration), which covered the entire answer process.

*Speech parameters* were extracted on the phoneme level using an automatic speech recognition system (Herms, 2016). The resulting transcripts included spoken units and the corresponding time codes in milliseconds and were used to derive the articulation rate, the number, and the mean duration of silent pauses. The *articulation rate* represented the total number of phonemes divided by the utterance duration excluding the total duration of silent pauses, the *number of pauses* reflected the total number of silent pauses in an utterance, and the *mean pause duration* was calculated from the total duration divided by the number of silent pauses.

*Physiological data* were recorded at a frequency of 128 Hz with a NeXus-10 Mark II from sensors attached to the volar surface of the distal phalanges of the left hand. While the heart rate (HR) signal was obtained at the trigger finger, the skin conductance response (SCR) was recorded at the middle and ring fingers. Data preparation involved the calculation of an individual baseline for each participant from values located in the preparation phase of about 5 min before starting the learning task. Recorded SCR and HR values were normalized to the individual baseline value and aggregated on mean values for events within each trial. Each event represented a subtask within the learning trial and was related to a screen change, i.e., the auditory presentation of a number sequence, the visual presentation of the symbol combination input, the response part of the presented symbol combination, a feedback on the given response, and finally the verbal recall of the memorized number sequence.

For obtaining *retention performance*, sum scores were calculated for all memorized symbol combinations and all correctly memorized symbol combinations, resulting in values ranging from 0 (neither combination memorized correctly) to 4 (all combinations memorized correctly). *Transfer performance* was obtained from the schema application task in terms of reaction times on the provided classifications as well as correct responses. The latter were adjusted for inherited errors from the retention task.

*Cognitive load scores* were derived from sum scores for each cognitive load facet, resulting in a maximum of 30 points for ICL and ECL and a maximum of 40 points for GCL. Subscales achieved satisfying internal consistencies of α = .831 for ICL, α = .708 for ECL, and α = .876 for GCL. In line with Conway et al. (2005), who reported a clear advantage of partial credit scoring procedures over all-or-nothing scoring procedures, the partial load score for the OSPAN was computed by awarding one point for each correctly recalled letter. Across the three test blocks, the task achieved an appropriate internal consistency of Cronbach’s α = .805.

## Results

### Cognitive load progression

Some datasets had to be excluded from data analysis due to missing values or the failure to meet the 85% accuracy criterion in the OSPAN^1^ task, a lack in language proficiency, or an observable violation of the instruction. Conditional growth curve models were computed to inspect progressions in primary and secondary task efficiency from a temporal perspective. Values for all relevant variables were *z*-standardized to obtain standardized β coefficients. Models were fit with restricted maximum-likelihood estimation and included time, condition, the OSPAN score, and the interaction between time and condition predictors as fixed effects aligned to the experimental task design. To take into account individual differences between participants in reaction to experimental variations, a time slope as well as subject-specific intercepts were considered as random effects and assumed to be correlated. In addition, time was computed as a logarithmic^2^ variable and included as a fixed effect. For evaluating model fit, the root mean squared error (RMSE) was obtained from a leave-one-out-cross-validation approach, and the conditional pseudo R^2^ for generalized linear mixed models was calculated.

#### Primary task efficiency

**Fig. 4:**
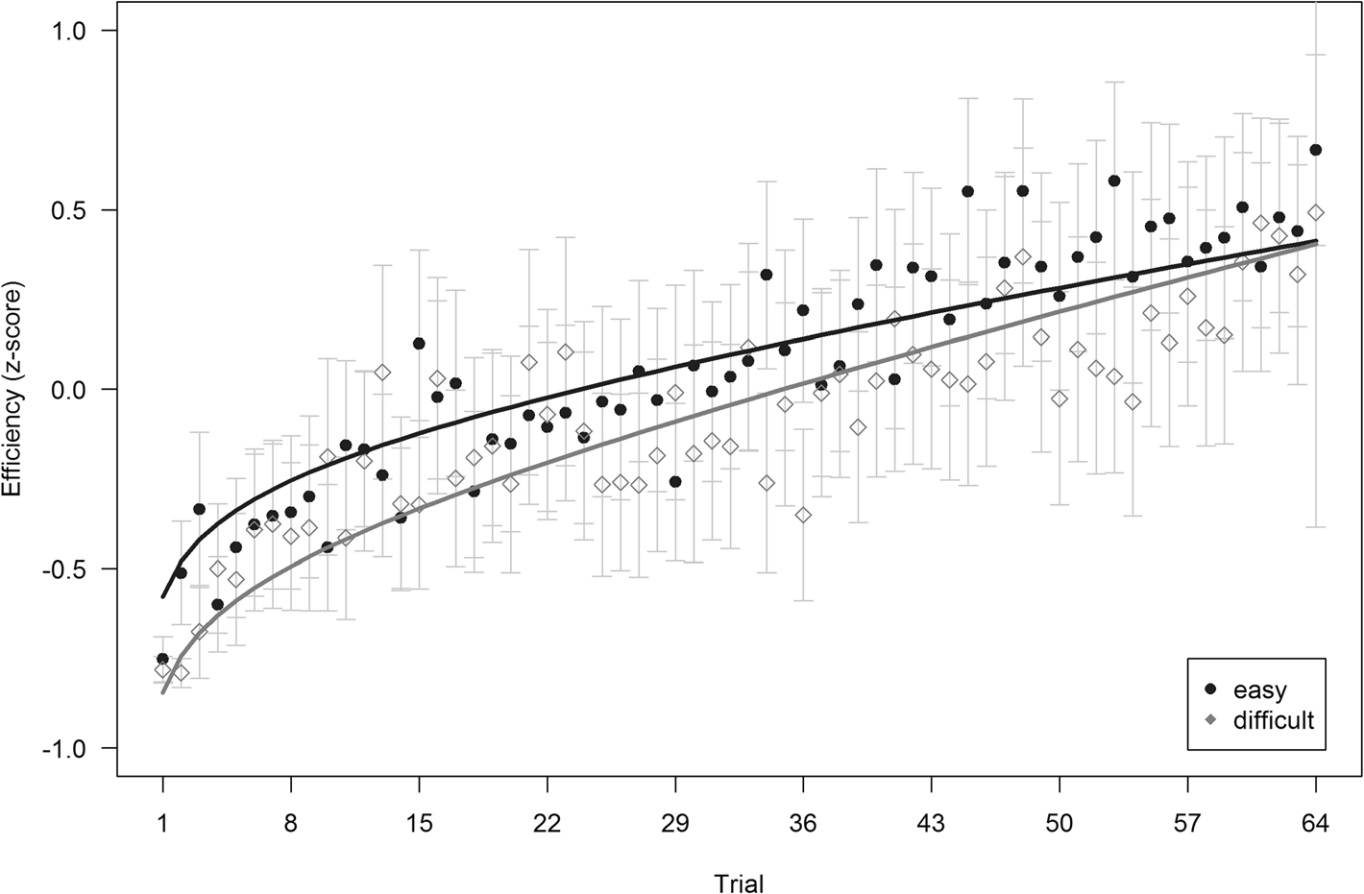
Changes in performance in primary task efficiency over the task. *Filled dots* and *empty rhombi* show empirical mean values per trial, *lines* indicate predicted mean values. Error bars indicate 95% confidence intervals from empirical observations

After exclusions, the analysis of primary task efficiency operated on *n* = 103 datasets. As indicated by Fig. 4, significant contributions resulted for the linear time predictor, β = .166, standard error (*SE*) = 0.030, *t*(683) = 5.609, *p* < .001, the logarithmic time predictor, β = 0.118, *SE* = 0.026, *t*(6385) = 4.573, *p* < .001, and condition, β = −.070, *SE* = 0.031, *t*(100) = − 2.220, *p* = .029. No significant interaction between time and condition could be observed, β = −.031, *SE* = 0.018, *t*(101) = − 1.726, *p* = .087. The model achieved an acceptable fit with RMSE = 0.936 and R^2^ = .197 and supports *hypothesis 1a*.

#### Secondary task efficiency

**Fig. 5:**
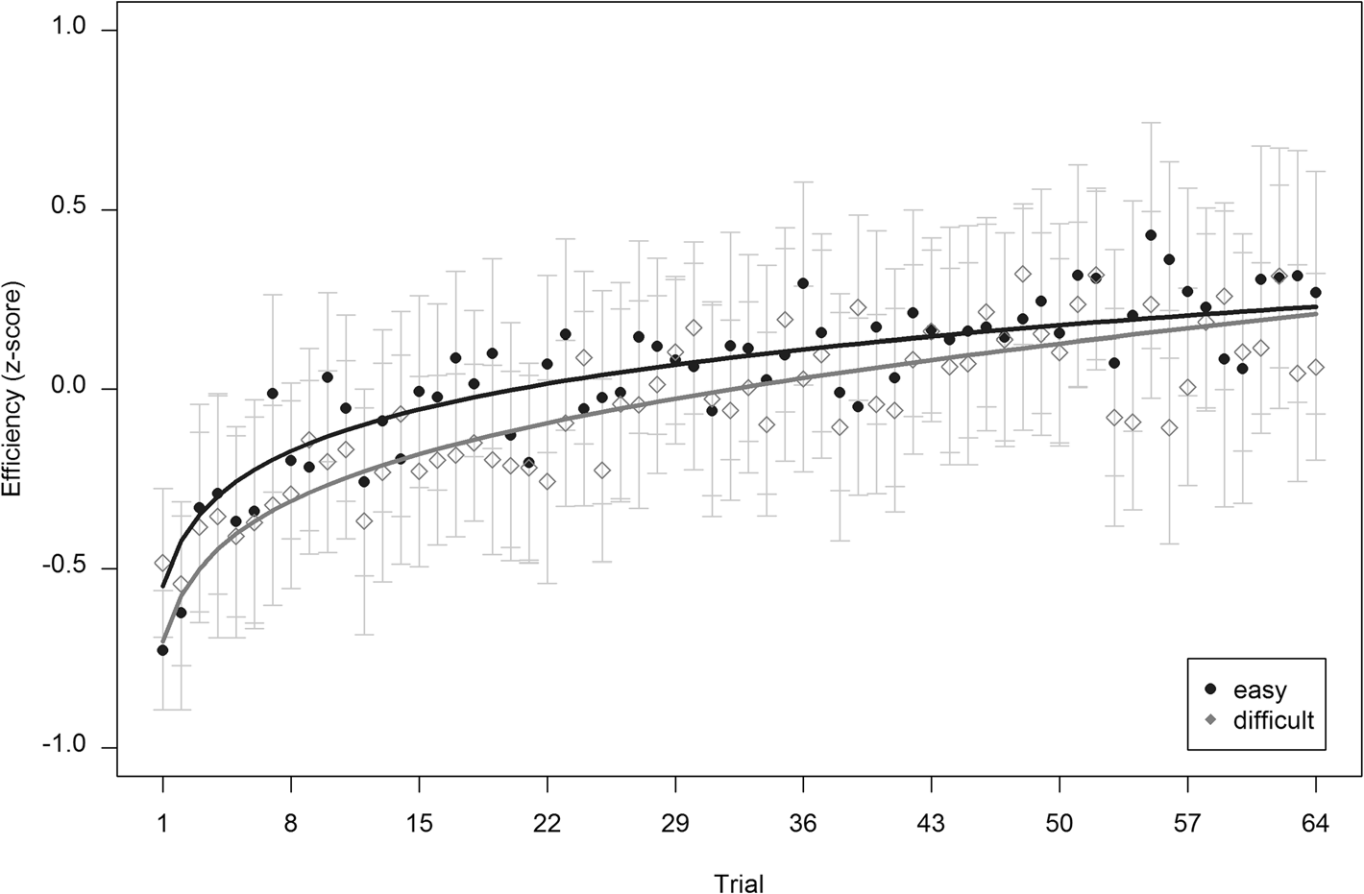
Changes in performance in secondary task efficiency over the task. *Filled dots* and *empty rhombi* show empirical mean values per trial, *lines* indicate predicted mean values. Error bars indicate 95% confidence intervals from empirical observations

When analyzing secondary task efficiency, one additional dataset had to be excluded from the sample due to high noise in the recorded speech signal, resulting in a subsample of *n* = 102 participants. Corresponding to the prevention of distraction effects on the primary task from an overly complex secondary task, participants achieved a predominantly high word accuracy (*M* = 0.95, *SD* = 0.15). As visually supported by Fig. 5, the results revealed an increasing secondary task performance efficiency over time as well. In more detail, standardized coefficients showed a significant logarithmic time predictor, β = .160, *SE* = 0.022, *t*(6323) = 7.392, *p* < .001, whereas no significance could be obtained for the linear time predictor, β = .029, *SE* = 0.028, *t*(361) = 1.022, *p* = .307, the effect of condition, β = −.044, *SE* = 0.062, *t*(99) = − 0.711, *p* = .479, and the interaction between time and condition, β = −.006, *SE* = 0.021, *t*(100) = − 0.271, *p* = .787. The overall model achieved a considerable fit with RMSE = 0.912 and R^2^ = .445 and supports *hypothesis 1a*.

#### Speech-related parameters

**Fig. 6:**
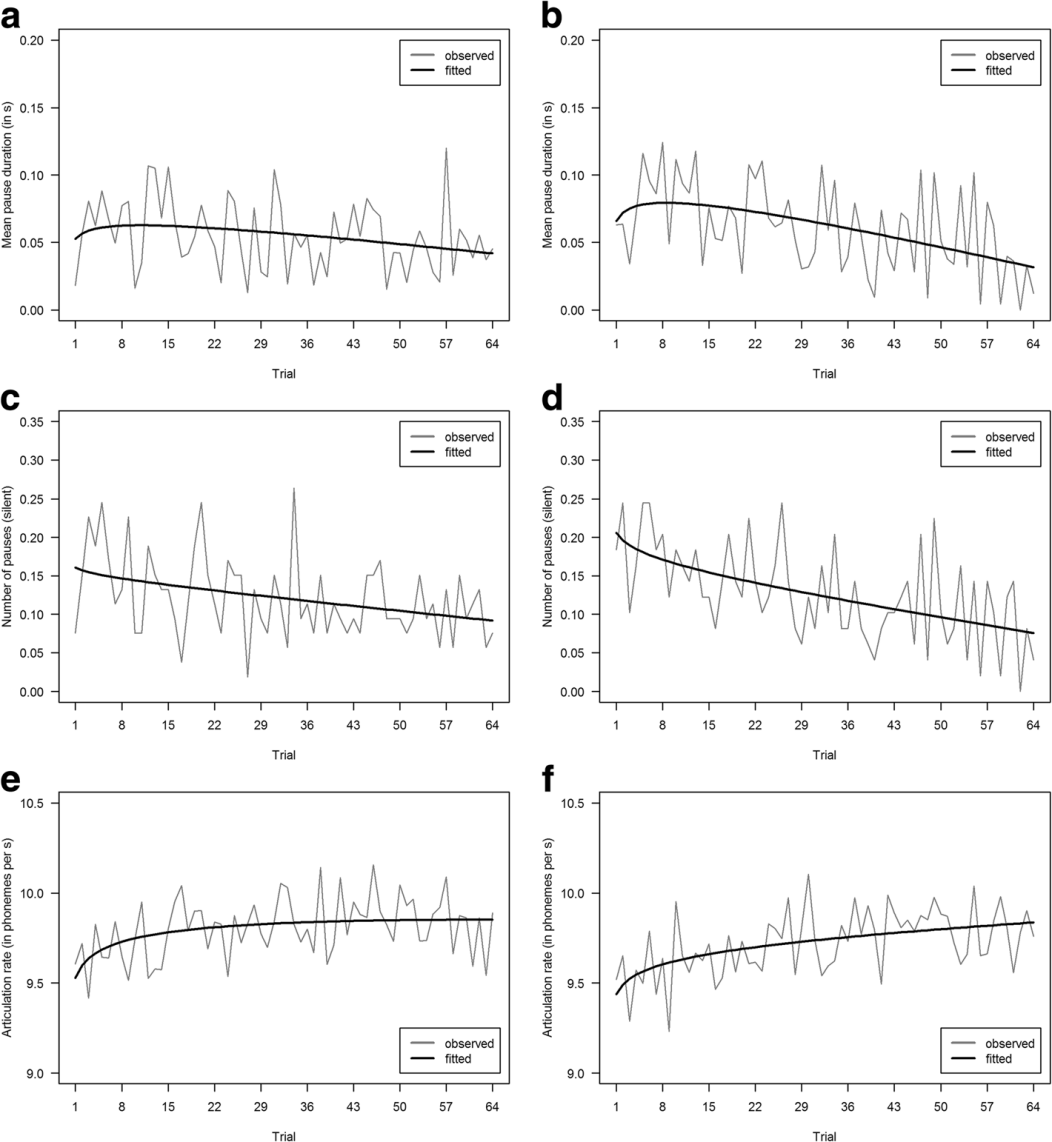
Observed and fitted logarithmic progressions of speech-related parameters computed per trial in easy (**a**, **c**, **e**) and difficult (**b**, **d**, **f**) conditions. Graphs in the *first row* refer to mean pause duration (total duration of silent pauses in seconds divided by the number of silent pauses). Graphs in the *second row* refer to the number of pauses (only silent pauses). Graphs in the *third row* refer to articulation rate (number of phonemes per second excluding pauses)

Corresponding to secondary task performance, results are based on *n* = 102 participants. Time series regressions with linear and logarithmic trend predictors, separated by conditions as depicted in Fig. 6, support decreases in cognitive load over time in terms of mean pause duration, R^2^_easy_ = .063 and R^2^_difficult_ = .216. In terms of number of pauses and articulation rate, the models achieved R^2^_easy_ = .131 and R^2^_difficult_ = .311 for number of pauses and R^2^_easy_ = .165 and R^2^_difficult_ = .268 for articulation rate. Although amounts of explained variance differ between measures and conditions, the overall trend supports *hypothesis 1a*.

#### Physiological parameters

**Fig. 7:**
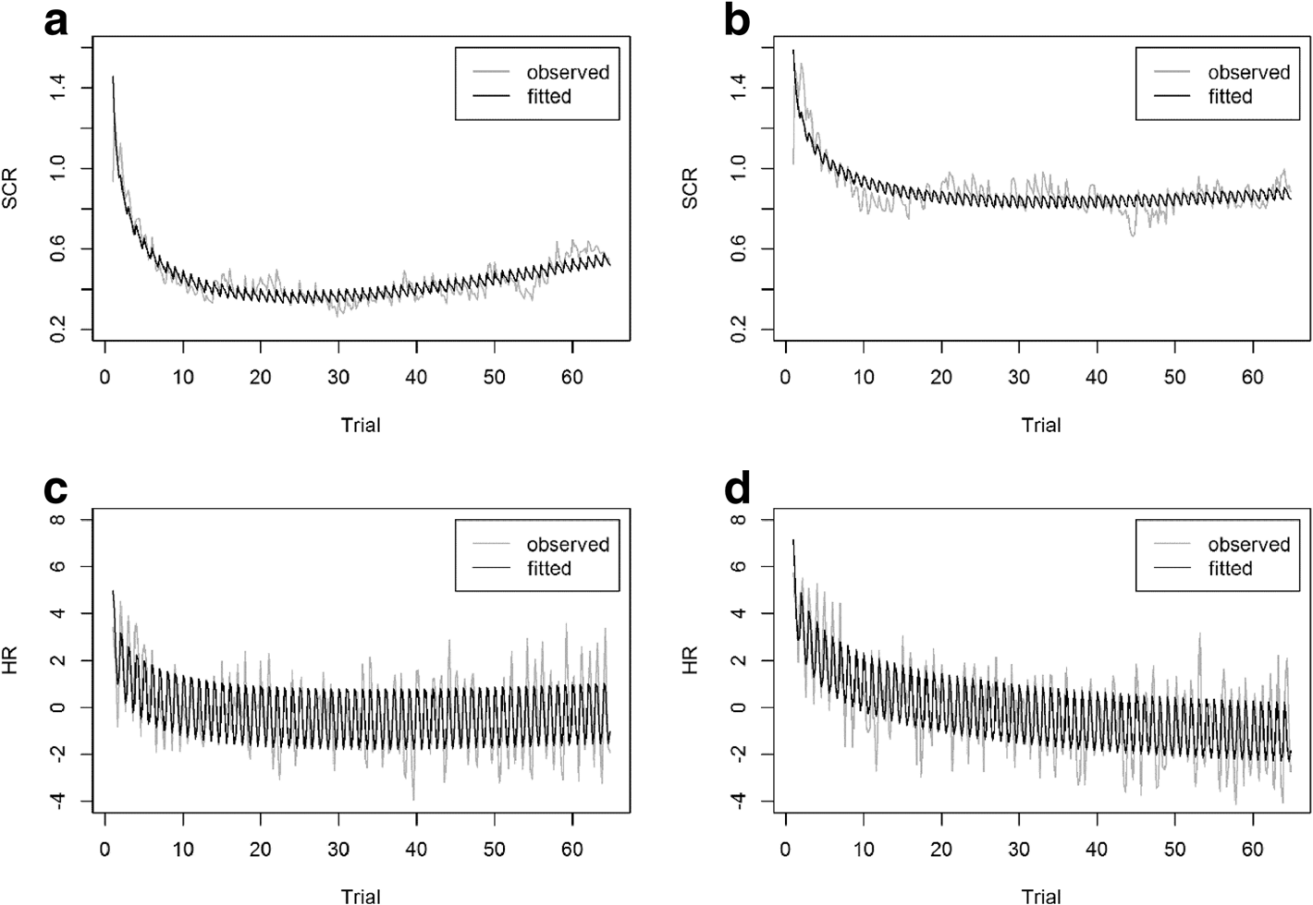
Observed and fitted logarithmic progressions of SCR and HR over time in easy (**a**, **c**) and difficult (**b**, **d**) conditions including both seasonal and trend components

Due to technical errors in the recorded data, two additional datasets had to be excluded from the analysis, resulting in a subsample of *n* = 101 participants. Assuming additive time series with trend and seasonal components, analyses indicate a decreasing trend of HR and SCR over the task and show a repetitive seasonal pattern across “subtasks” within each trial. Logarithmic time series regression models, including linear and nonlinear trend predictors as well as seasonal predictors, achieved R^2^_easy_ = .847 and R^2^_difficult_ = .672 for SCR, whereas for HR an R^2^_easy_ = .590 and R^2^_difficult_ = .643 could be obtained. Inspecting the seasonal component in more detail, following an initial increase while the sequence of numbers was presented auditorily, a decreasing progression over the symbol presentation, symbol response, and symbol feedback could be observed. However, in the final step of verbally recalling the memorized numbers, physiological response parameters increased again. Fig. 7 displays the respective observed and fitted logarithmic progression curves, which strongly support *hypothesis 1a*.

### Retention and transfer performance

#### Retention performance

Results for both retention and transfer performance are based on *n* = 103 participants. In terms of retention performance, in line with *hypothesis 2a*, significantly fewer correctly recalled symbol combinations could be observed in the difficult conditions, *t*(96.53) = 4.72, *p* < .001, *d* = − 0.92. No significant differences between conditions regarding the amount of totally recalled symbol combinations resulted, *t*(100.99) = − 0.08, *p* = .937, *d* = 0.02. A power^3^ level of 1 – β = .71 was achieved for α = .05 and *d* = 0.5.

#### Transfer performance

As already indicated from the descriptive values in Table 2, results revealed significantly faster responses in the difficult condition, *F*(1, 101) = 5.59, *p* = .020, η_p_^2^ = .05, reversing the pattern postulated in *hypothesis 2b*. The significant increase in reaction time with increasing distortion, *F*(3, 303) = 15.13, *p* < .001, η_p_^2^ = .13, partially supports *hypothesis 2c*. A significant interaction between both factors did not result, *F*(3, 303) = 0.60, *p* = .614, η_p_^2^ = .01, 1 – β = 1.0 for α = .05 and *f* = .25. Post hoc pairwise comparisons using Tukey’s honest significant difference (HSD; Maxwell, 1980) indicate significant differences in reaction time between the prototypical level and all levels of distortion (all *p*’s < .05) as well as the distortion levels 30 and 90 (*p* < .001). After correction for inherited errors, no significant differences result in correct responses between conditions, *F*(1, 101) = 0.99, *p* = .323, η_p_^2^ = .01, 1 – β = .941, and levels of distortion, *F*(3, 303) = 1.42, *p* = .237, η_p_^2^ = .01, 1 – β = 1.0. Likewise, no significant interaction between both factors could be detected, *F*(3, 303) = 0.91, *p* = .436, η_p_^2^ = .01, 1 – β = 1.0. All reported power levels relate to α = .05 and *f* = .25. In general, with around 70% in each level of distortion, a high frequency of correct responses was observable (see Table 2), hinting at a rather stable and robust application of previously acquired schemata.

**Table 2:** Descriptive values of correct responses and reaction time for levels of distortion in conditions

Dist.	RT_easy_		RT_difficult_		CR_easy_		CR_difficult_	
	*M*	*SD*	*M*	*SD*	*M*	*SD*	*M*	*SD*
0	2571.14	1079.33	2261.58	1010.11	0.75	0.44	0.71	0.46
30	2692.68	1168.68	2381.37	1116.81	0.70	0.46	0.70	0.46
60	2670.05	1208.94	2485.99	1091.26	0.72	0.45	0.69	0.46
90	2776.47	1178.77	2519.48	1097.61	0.72	0.45	0.68	0.47

*Dist.* severity of distortion in %, *RT* reaction time, *CR* correct responses (min = 0, max = 1), *easy* easy condition, *difficult* difficult condition

### Subjective cognitive load ratings

Contrary to *hypothesis 1b*, no significant differences between easy and difficult conditions appeared for ICL, *t*(90.76) = − 1.13, *p* = .225, *d* = 0.306, and ECL, *t*(97.57) = − 1.22, *p* = .226, *d* = 0.242, although descriptive values pointed towards lower scores for the easy condition. By contrast, significantly higher ratings in the easy condition resulted for GCL, *t*(98.77) = 2.87, *p* = .005, *d* = − 0.568, which directly reverses the pattern postulated in *hypothesis 1b*. According to Cohen (1988), the effect amounts to a medium size. Analyses achieved a power of 1 – β = .709 for α = .05 and *d* = 0.5.

## Discussion

This study provided insight into the progression and interaction of the outlined facets of cognitive load by applying a multi-method approach to cognitive load assessment. In line with the postulated hypothesis, performance, speech, and physiological parameters indicated a logarithmically decreasing level of cognitive load over the task, hinting at increasing progress in schema acquisition. The subjective ratings did not support the initial assumptions, but support arises for the decrease in retention performance with higher levels of task complexity. For transfer performance, the stated hypothesis on increased transfer demands was partly confirmed in terms of reaction times, while evidence indicated a reversed pattern for task complexity in this case.

In addition, physiological measures revealed a repetitive seasonal pattern across the subtask routines for SCR and HR: during the presentation of the auditory stimulus at the outset of each trial, an increase was observable. It was followed by a decrease related to the visual presentation of the symbol combination, the motor response selection, and the visual feedback screen. As soon as the verbal response on the initially presented digit sequence was requested, increasing levels in both physiological measures again resulted. This evidence suggests higher levels of cognitive load imposed by the auditory-verbal compared to the visual-motor stimulus-response combination due to the observable initial and final increase in the emerging signal. Support for this assumption arises from Posner, Nissen, and Klein (1976), who state the dominance and increased familiarity of the visual modality compared to other modalities. Another potential clarification suggests increased perceptual load (Lavie, 2010) in the auditory-verbal secondary task, imposed by the additional visual stimulus from the speaker and speech bubble symbols shown on the screen.

Approaching the obtained pattern in the subjective cognitive load ratings, at least on a descriptive level participants reported lower ICL and ECL. On the one hand, these scores might have lacked statistical significance due to the reported insufficient power level of about 71%. On the other hand, as secondary task requirements did not differ between conditions, the absence of considerable differences in ECL is indeed plausible. Moreover, adding just one symbol to the combination might not have resulted in extensive increases in task complexity, potentially explaining the absence of significant differences in ICL. The reversed pattern for GCL could have originated in the particular formulation of the related questions, which emphasizes the subjective impression of understanding and knowledge acquisition. This might have been higher in the easy condition. The authors (Leppink et al., 2013) also discuss this issue in a more recent publication that applies this questionnaire (Leppink, Paas, Van Gog, Van der Vleuten, & Van Merriënboer, 2014). They attribute the lack of meaningful results for GCL to the substantial redundancy between GCL and ICL and take this as further evidence for the initially outlined re-reduction of the three-factor model. By contrast, a more recently developed cognitive load questionnaire by Klepsch, Schmitz, and Seufert (2017) addresses this issue by including the effort component more explicitly in the GCL facet and advocates the existing three-factor model from an assessment perspective. As the authors claim its applicability for a wider range of learning contexts and domains, using this questionnaire instead would be a valuable extension in future studies.

Regarding the schema application task, faster response times in the difficult condition compared to the easy condition hint at higher investments of mental resources with higher levels of task complexity. This explanation corresponds well with results on contextual interference (de Croock, Van Merriënboer, & Paas, 1998; Van Merriënboer et al., 2006) which report that learners can achieve a more robust and stable transfer performance under conditions that disable fast and easy skill acquisition. Additional support arises from evidence on desirable difficulties in learning situations (Bjork & Bjork, 2011). For instance, when learners must cope with interleaved tasks, they need to maintain sustained engagement of cognitive resources, which fosters performance. The observed increase in response times with increasing distortion may result due to the requirement of additional cognitive operations, as distorted symbols demand the identification of the underlying symbol category before the judgement.

### Implications

The pattern of evidence supports a temporal extension of the CLT framework and reveals a logarithmically decreasing cognitive load progression with increasing schema acquisition. Moreover, results indicate the benefit of an automatic detection of the current level of cognitive load in speech parameters. This could be of value for realizing adaptive user interfaces in digital learning contexts that bear the ability to adjust task complexity and instructional guidance to learners’ needs and preferences. A particularly promising field of application comprises foreign language learning, where spoken interaction during the learning process constitutes an essential pre-requisite to shape language skills. User-aligned instructional aids within a considered language training program might then entail additional explanations or calming feedback if states of high load are detected by extended pausing or low articulation frequencies.

### Limitations

A potential limitation arises from the differing symbol complexity, since a visually dominating appearance like a star, with salient edges and corners, could foster and speed up schema acquisition and thus benefit symbol recall. More detailed analyses of times spent on drawing different symbols in the retention questionnaire by mouse tracking or gaze pattern analyses might enlighten this issue in future research. With reference to the non-prototypical symbols used for the schema application task, although there was a high level of consensus in the pre-study regarding the correct match to the underlying prototype, subtle differences still could have persisted and influenced the findings in the main task.

The lack of significant differences between conditions in secondary task efficiency could have resulted from task order ambiguities. Although participants were instructed to give equal weight to both tasks, the secondary task occurred first in order and could have been regarded as more easy and familiar. For this reason, participants might have been motivated to prioritize this task instead of the primary task and assign only free resources to the primary task. In consequence, the easy task condition would achieve better results in primary task efficiency, whereas secondary task performance would stay unaffected by task complexity. The choice of keeping this fixed presentation order across all trials and participants is aligned to the original task procedure reported for automated complex span tasks (Redick et al., 2012; Unsworth et al., 2005). Using a counterbalanced presentation order instead could result in more balanced weighting of the priority of both tasks in future studies. A further valuable extension could address the relationship between both tasks more explicitly to obtain insights into participants’ strategies of cognitive resource distribution. The additional use of cognitive models (Anderson, 1983) that compare both task order strategies could support the clarification of distinct effects of task order prioritization.

### Future research

Prospective research should monitor learners’ focus on presented symbols or alternative learning material by inspecting gaze behavior in combination with pupil dilation, as suggested by Foroughi, Sibley, and Coyne (2017) and Mitra, McNeal, and Bondell (2017). Another promising extension incorporates the transfer of the obtained patterns and mechanisms to more applied task settings in a different task domain like motor learning. In this domain, an additional step could involve the inclusion of a spatial dimension or the use of animated stimuli or motor sequences, and distinct audio-verbal secondary tasks with a different task order are suitable as well. A further interesting extension takes evidence on modality compatibility in dual-task settings (Hazeltine et al., 2006) into account and inspects the use of incompatible content-modality matchings.

## Conclusion

The study applied a multi-measure framework of cognitive load assessment to gain further insights into the temporal progression of cognitive load during schema acquisition. Results replicate the logarithmic pattern of change over the task observed in prior research and reveal influences of task complexity and situational constraints. In summary, the promising approach holds value to address existing research gaps in cognitive load research and gain better insights into changing demands from schema acquisition in learning settings.

## Abbreviations

ANOVA: Analysis of variance
CLT: Cognitive load theory
ECL: Extraneous cognitive load
GCL: Germane cognitive load
HR: Heart rate
HSD: Honest significant difference
ICL: Intrinsic cognitive load
OSPAN: Operation span task
RMSE: Root mean squared error
SCR: Skin conductance response
SE: Standard error
STEM: Science, Technology, Engineering, and Mathematics

## Footnotes

1 This exclusion involved seven participants, which corresponds to about 6% of the sample. For comparison, Unsworth et al. (2005) report exclusion rates of 15%.

2 In line with Wirzberger et al. (2017), alternative linear and quadratic progression models were tested as well, but not reported due to the confirmed advantage of the logarithmic model and the lack of benefit for the focus of this study.

3 Power analyses refer to theoretically assumed population effect sizes to provide a broader informative value for the reasoning about non-significant results (O'Keefe, 2007).
